# Supplementary material for: From sequence to enzyme mechanism using multi-label machine learning
Source: BMC Bioinformatics. 2014 May 19;15:150. doi: 10.1186/1471-2105-15-150 (PMC4229970; doi:10.1186/1471-2105-15-150)
Supplement: Additional file 2 — Java code of ml2db. Additional file ml2db_code.tar.gz contains the Java source code to run the multi-label machine learning experiments and save the results to database. The code’s Javadoc is included. [file 1471-2105-15-150-S2.zip › additional file 2/ml2db/ml2db/doc/main/ml2db/graphviz/GraphVizPrinter.html]

GraphVizPrinter


JavaScript is disabled on your browser.


- Overview
- Package
- Class
- Use
- Tree
- Deprecated
- Index
- Help

- Prev Class
- Next Class

- Frames
- No Frames

- All Classes

- Summary:
- Nested |
- Field |
- Constr |
- Method

- Detail:
- Field |
- Constr |
- Method


main.ml2db.graphviz

## Class GraphVizPrinter

- java.lang.Object
- - main.ml2db.graphviz.GraphVizPrinter

- ---

    

  ```
  public class GraphVizPrinter
  extends java.lang.Object
  ```

  Class

  Version:
  :   27 Jul 2011

  Author:
  :   Luna De Ferrari ldf(at)st-andrews.ac.uk

- - ### Constructor Summary

    Constructors

    Constructor and Description

    `GraphVizPrinter()`
  - ### Method Summary

    Methods

    | Modifier and Type | Method and Description |
    | --- | --- |
    | `static void` | `main(java.lang.String[] args)` |
    | `static void` | `printNeighboursForGraphviz(java.lang.String filePath, NeighbourhoodNetwork network, boolean inColour)` |
    | `static void` | `run()` |
    | `static void` | `runFromSerialised()` |

    - ### Methods inherited from class java.lang.Object

      `equals, getClass, hashCode, notify, notifyAll, toString, wait, wait, wait`

- - ### Constructor Detail


    - #### GraphVizPrinter

      ```
      public GraphVizPrinter()
      ```
  - ### Method Detail


    - #### main

      ```
      public static void main(java.lang.String[] args)
      ```


    - #### printNeighboursForGraphviz

      ```
      public static void printNeighboursForGraphviz(java.lang.String filePath,
                                    NeighbourhoodNetwork network,
                                    boolean inColour)
      ```


    - #### run

      ```
      public static void run()
      ```


    - #### runFromSerialised

      ```
      public static void runFromSerialised()
      ```


- Overview
- Package
- Class
- Use
- Tree
- Deprecated
- Index
- Help

- Prev Class
- Next Class

- Frames
- No Frames

- All Classes

- Summary:
- Nested |
- Field |
- Constr |
- Method

- Detail:
- Field |
- Constr |
- Method
